# Range of Resection in Endometrial Cancer—Clinical Issues of Made-to-Measure Surgery

**DOI:** 10.3390/cancers16101848

**Published:** 2024-05-11

**Authors:** Agnieszka Horala, Sebastian Szubert, Ewa Nowak-Markwitz

**Affiliations:** Division of Gynaecological Oncology, Department of Gynaecology, Poznan University of Medical Sciences, 61-701 Poznan, Poland; sszubert@ump.edu.pl (S.S.); ewamarkwitz@ump.edu.pl (E.N.-M.)

**Keywords:** endometrial cancer, uterine cancer, endometrial neoplasms, gynecologic cancer, surgery, lymphadenectomy, sentinel lymph node, molecular subtypes

## Abstract

**Simple Summary:**

For a couple of decades, the morbidity rates for endometrial cancer (EC) have been on a constant rise. Surgery is the cornerstone in the management of this disease and may be performed for curative, staging, or palliative purposes. The type of hysterectomy, the role and extent of lymphadenectomy, the procedure of the sentinel lymph node mapping, and cytoreductive surgery in advanced or recurrent EC are discussed in detail. Most recently, the introduction of the molecular classification has changed the scene in EC treatment, and its impact on choosing the surgical strategy is outlined. This narrative review focuses on the intricacies of surgical management of EC and aims at summarizing the available literature on the subject providing an up-to-date clinical guide.

**Abstract:**

Endometrial cancer (EC) poses a significant health issue among women, and its incidence has been rising for a couple of decades. Surgery remains its principal treatment method and may have a curative, staging, or palliative aim. The type and extent of surgery depends on many factors, and the risks and benefits should be carefully weighed. While simple hysterectomy might be sufficient in early stage EC, modified-radical hysterectomy is sometimes indicated. In advanced disease, the evidence suggests that, similarly to ovarian cancer, optimal cytoreduction improves survival rate. The role of lymphadenectomy in EC patients has long been a controversial issue. The rationale for systematic lymphadenectomy and the procedure of the sentinel lymph node biopsy are thoroughly discussed. Finally, the impact of the molecular classification and new International Federation of Gynecology and Obstetrics (FIGO) staging system on EC treatment is outlined. Due to the increasing knowledge on the pathology and molecular features of EC, as well as the new advances in the adjuvant therapies, the surgical management of EC has become more complex. In the modern approach, it is essential to adjust the extent of the surgery to a specific patient, ensuring an optimal, made-to-measure personalized surgery. This narrative review focuses on the intricacies of surgical management of EC and aims at summarizing the available literature on the subject, providing an up-to-date clinical guide.

## 1. Introduction

For a couple of decades, the morbidity rates for endometrial cancer (EC) have been on a constant rise [1]. Among the main factors responsible for this trend, obesity and longer life expectancy can be listed; therefore, further increase in EC rates is likely. In general, primary treatment of EC is surgical with adjuvant radiotherapy and/or chemotherapy and, most recently, immunotherapy, which has emerged as a new treatment modality, especially for tumors with mismatch repair (MMR) deficiency. In the recent years, there have been major advances in understanding the pathologic and molecular features of EC, and four molecular types were distinguished [2], depending on the mutations present in the tumor. Although the differences in clinical behavior of various molecular EC types are not yet entirely clear, the discovery has already immensely affected risk stratification in EC and thus the therapeutic options, contributing to the update of EC staging by FIGO that was published in 2023 [3].

Surgery is the cornerstone in the management of EC, and currently more than 90% of newly diagnosed ECs are treated with surgery [4], which may be performed in all stages of the disease. In general, surgery in EC has several goals that differ according to the clinical situation. The main goal of surgery is the radical removal of the disease (primary tumor and metastases) with a curative intent. In addition, surgery is required for staging to identify risk factors and adequately qualify the patients for adjuvant treatment. However, in selected situations, the main aim of surgery may be palliation, e.g., to stop active bleeding.

Recently, minimally invasive surgery (MIS), either laparoscopic or robotic, has become the standard in EC treatment and should always be considered. A number of trials showed that MIS is associated with shorter hospital stays, decreased need for post-operative analgesia, faster recovery, and improved quality of life [5,6]. Advances in the technical possibilities and surgeons’ skills made most of the oncological surgical procedures possible (e.g., pelvic lymphadenectomy, radical resection of the parametria). However, there was a concern if minimally invasive approaches provide oncological safety. The first turning point to demonstrate non-inferiority of minimally invasive techniques in EC treatment was the publication of the results of a randomized prospective clinical trial (GOG-222, LAP2) comparing the outcomes of surgical staging of EC by laparotomy versus laparoscopy [7,8]. The trial reported comparable oncological safety for both techniques (three-year recurrence rates of 11.2% for laparoscopy and 10.2% for laparotomy and five-year OS of 89.8% in both groups). Similar results were found in the Laparoscopic Approach to Cancer of the Endometrium (LACE) trial, limited to patients with apparent stage I EC of endometrioid histology, which compared the outcomes of laparoscopic vs. abdominal hysterectomy with/without lymphadenectomy and revealed no differences in DFS and OS [9]. Long-term survival outcomes were comparable for minimally invasive surgery and open surgery in EC patients with high-risk histology [10].

Robotic surgery, although more costly, may facilitate complex surgical dissection, e.g., para-aortic lymphadenectomy. Robotic surgery has the same benefits as laparoscopy, similar operating time (both take longer than laparotomy), and additionally was reported to cause less blood loss and a smaller conversion to laparotomy rate (9.9% for laparoscopic and 4.9% for robotic hysterectomies, respectively) [11].

The data on vaginal hysterectomy in EC is limited, and its application is restricted due to the inability to inspect the abdominal cavity and stage the patient, as well as due to difficulties in the removal of the adnexa. It may be considered in selected patients with early stage EC with comorbidities that limit the extent of the Trendelenburg position and pneumoperitoneum, which are necessary for laparoscopic and robotic surgery [12].

Current evidence shows that all types of surgery (open, laparoscopic, robotic) have similar oncologic outcomes, and thus minimally invasive surgery is the preferred surgical approach, including patients with high-risk EC. Still some uncertainties remain regarding the appropriate surgical treatment of different stages and histopathological types of EC. The aim of this narrative review is to summarize the current knowledge, identify ambiguities, and indicate further directions of research in the field of surgical management of EC. In this review, data concerning surgery depending on stage of the disease and histopathological type of EC is provided. In the text, we make references to the EC stage according to the American Joint Committee on Cancer (AJCC) tumor-node-metastasis (TNM) classification and the Federation of Gynecology and Obstetrics (FIGO). Furthermore, the surgical treatment in selected clinical situations is discussed. Last but not least, in this review, we discuss how the molecular profiling of EC may influence surgical treatment.

## 2. Type of Hysterectomy in Endometrial Cancer Confined to the Uterus (T1a-b/FIGO I)

In the case of EC limited to the uterus without cervical involvement, a simple hysterectomy ensures adequate surgical margins, and it seems that there is no need for parametrectomy in these cases. Signorelli et al., in a randomized controlled trial comparing modified radical hysterectomy to simple total extrafascial hysterectomy in the treatment of stage I EC, showed similar locoregional control and survival [13].

Simple total hysterectomy can be performed using intrafascial or extrafascial excision. The former is easier and quicker, while the extrafascial excision requires training in ureter mobilization. Extrafascial hysterectomy enables removal of the cervix with surrounding connective tissue (pericervical fascia). In the case of intrafascial hysterectomy, the excision is performed through pericervical fascia; therefore, small portions of the cervix remain in situ. In the case of endometrial cancer confined to the uterine corpus, both intra- and extrafascial hysterectomy ensures oncological free margins [14]. However, considering the ontogenetic cancer field theory, it would be reasonable to remove the entire uterus with intact cervix to obtain the removal of one entire Müllerian subcompartment [15,16,17]. Moreover, in the case of unrecognized cervical stromal invasion prior to surgery, extrafascial excision would ensure adequate surgical margin. To the best of our knowledge, it was never evaluated in a clinical trial whether intra- or extrafascial total hysterectomy is more appropriate for early stage EC. Both the European Society of Gynaecological Oncology (ESGO), the European Society for Radiotherapy and Oncology (ESTRO), and the European Society of Pathology (ESP) and The National Comprehensive Cancer Network (NCCN) recommend “total hysterectomy” for apparent stage I endometrial cancer without specifying between intra- and extrafascial techniques [18,19].

Another point in the surgical management of early stage EC is the length of vaginal resection. This issue was evaluated only in retrospective trials that yielded opposite results. Vizza et al. showed that the length of the vaginal cuff is not related to the risk of recurrence in low-risk EC [20]. On the contrary, in the study by Arndt-Miercke et al., the authors found increased risk of recurrence and impaired overall survival (OS) in patients without the vaginal cuff removal [21]. The extent of the vaginal cuff excision is related to the type of hysterectomy and parametrial resection as it is generally difficult to excise the vaginal cuff without performing at least partial parametrectomy. As it was stated before, prospective trials showed no benefit of parametrial resection in stage I EC, and therefore there are also no studies supporting vaginal cuff excision in stage I EC.

The estimated rate of omental micrometastases in apparent stage I endometrioid EC is 3.0% and correlates with extrauterine spread to other sites [22,23]; therefore, routine omentectomy in this group of patients is not recommended [18]. It is worth noticing that metastases in the omentum change the staging to IVA according to FIGO 2023 classification [3]. Positive peritoneal cytology is present in nearly 10% of the patients with apparent stage I EC [22] and correlates with poor prognostic factors, such as grade 3 and myometrial invasion >50% and with poor survival [24] Some studies suggested that positive peritoneal washings may result from uterine manipulation [25]. Nevertheless, it was removed from the FIGO staging system in the 2009 update [26], and it is unclear whether it should have a role in qualifying patients for adjuvant therapy [9].

Taking into account all the evidence, the standard surgery for managing clinical stage I endometrioid EC is simple total hysterectomy with bilateral salpingo-oophorectomy without vaginal cuff resection, preferably by a minimally invasive approach [18]. Although it is not entirely clear whether the surgery should by performed by a extrafascial or intrafascial technique, the extrafascial hysterectomy is the most studied technique for hysterectomy in early stage EC and it ensures total removal of the uterus.

## 3. Type of Hysterectomy in Endometrial Cancer with Cervical Involvement (T2/FIGO II)

EC involving cervical stroma constitutes about 7% of ECs [27]. This group of patients are at a higher risk of parametrial involvement, which was found in around 10% of patients with cervical involvement and strongly correlated with other high-risk features such as lymph node involvement, ovarian metastases, and lympho-vascular space invasion (LVSI) [28,29]. Due to the high risk of parametrial involvement in T2 EC, parametrectomy should be considered. However, in the meta-analysis by Liu et al., radical hysterectomy failed to have an impact on the local recurrence rate, disease-free survival (DFS), and OS when compared to simple hysterectomy [30]. Similar results were obtained by Nasioudis et al., who evaluated over seven thousand patients with EC involving cervical stroma [31]. The lack of difference between simple and radical hysterectomy in the case of T2 EC patients may be attributed to the common use of adjuvant radiation, which provides a high rate of oncological clearance of parametrial micrometastases.

One of the principal rules in oncological surgery is to excise the tumor with free margins. In the case of uterine cervix cancerous infiltration, it may be challenging to achieve tumor-free margins with a simple hysterectomy. Furthermore, due to a likely disturbance of the anatomy by the bulky cervix, the risk of ureter injury is increased. For this reason, radical hysterectomy should be considered when the infiltration of the cervix is very close or reaching the pericervical fascia [18], and if so, type A or type B Querleu–Morrow [32] hysterectomy is usually sufficient.

Cervical stromal infiltration by EC should be distinguished with a parameter known as lower uterine segment involvement (LUSI). LUSI was found to correlate with metastases in the lymph nodes [33]. However, in the absence of lymph node involvement, it lacked prognostic significance and thus is not recommended to be used as an indication for adjuvant therapy [34,35].

## 4. Surgical Management of Advanced EC (FIGO Stages III–IV) of All Histological Types

Several studies indicated that surgical removal of all the cancerous lesions in advanced EC improves survival [36,37]. In a meta-analysis comprising over 3000 patients with EC FIGO stage IIIA-IVB (according to FIGO 2009 classification) of various histological subtypes, leaving no gross residual disease was possible in 52.1% of cases, and performing optimal cytoreduction defined as <1 cm of gross residual disease in 75.0%. Interestingly, the success rate of cytoreduction did not vary significantly by histology [36]. Most importantly, it was found that any gross residual disease versus no gross residual disease was associated with worse survival outcomes—the reported hazard ratios for progression-free survival (PFS) and OS were 2.16 and 2.57, respectively. Similar results were obtained for the difference between <1 cm and ≥1 cm gross residual disease, thus indicating the crucial role of optimal cytoreductive surgery in advanced EC treatment [36]. Therefore, the primary aim of surgery in EC stages III-IV is maximal cytoreduction, and complete or at least optimal (leaving <1 cm residual disease) debulking should be performed whenever possible with acceptable morbidity, taking into account the need for subsequent adjuvant therapy [18]. However, when primary optimal debulking is not feasible, alternative treatment options should be considered to avoid excessive morbidity [38]. In the study by Albright et al., the success rate for maximal/optimal cytoreduction negatively correlated with stage of EC [36]. The likelihood of obtaining maximal/optimal cytoreduction was also reported to be negatively associated with spread in the upper abdomen, involvement of three or more anatomic sites, and obesity [38,39,40].

Clinically, EC substages FIGO IIIA-IVB can have quite different presentation and require different surgical handling. Stage FIGO IIIA means spread to the surface of the uterus and/or adnexa. In that case, a simple hysterectomy with bilateral salpingoophorectomy is sufficient to achieve free margins. However, adnexal involvement is often associated with the peritoneal type of spread, and taking this into account, omentectomy, careful peritoneal inspection, and biopsies seem reasonable.

Stage IIIB2 was recently distinguished in FIGO 2023 classification [3]. Previously, patients with peritoneal metastases were classified as stage FIGO IVB. Stage IIIB2 ECs share similar characteristics to stage IIIB/IIIC ovarian cancers with dissemination to pelvic peritoneum. Total hysterectomy with bilateral salpingo-oophorectomy and pelvic peritonectomy usually ensure cytoreduction to no macroscopic residual disease in these cases.

FIGO stage IIIB1 and IVA (involvement of the bladder and/or rectal mucosa) represent locally advanced EC. It is worth noting that while infiltration of the bladder or rectal wall in not uncommon, it hardly ever reaches the mucosa. Should this be the case, it often results in the formation of a fistula. These two substages are scarce in the clinical practice (reported rates of stage IIIB are 2.3–6.2% and of stage IVA 1.5–5.9%), as metastases in the retroperitoneal lymph nodes or distant metastases often coexist with locally advanced EC [38,41]. The high risk of metastatic disease puts into question the rationale for aggressive pelvic surgery. Parametrial involvement is usually microscopic and diagnosed after hysterectomy. In clinical practice, occult parametrial and vaginal infiltration are challenging, as such cases would require extensive surgery associated with a significant risk of the bladder and ureter resection. For this reason, many of the FIGO stage IIIB patients are not good candidates for primary surgery.

Similar limitations apply to EC stage IVA where optimal cytoreduction usually requires some form of pelvic exenteration. However, in these cases, the risk of occult metastatic disease and major postoperative complications has to be carefully balanced against the chance for achieving complete cytoreduction [42]. There are limited data on primary pelvic exenterations in EC, and most papers report treatment outcomes for recurrent disease or for different types of advanced gynecologic malignancies jointly, and it is not certain if the data can be extrapolated to EC [42,43,44]. In the series of 40 EC patients that underwent pelvic exenteration due to primary advanced (8%) or recurrent (92%) disease, the OS rates were substantially higher for those operated with a curative aim as opposed to those undergoing palliative surgery (72.6% vs. 19.1% at 5 years and 59.4% vs. 0% at 10 years, respectively), showing that pelvic exenteration can provide long-term survival in selected patients [44]. The indications for primary pelvic exenteration are usually limited to symptomatic cases with vesico-vaginal or recto-vaginal fistulas, in which case, it can be offered as a radical treatment as opposed to palliative surgery (e.g., nephrostomy, colostomy).

Stage IVB according to the FIGO 2009 classification was a non-uniform group of patients encompassing, on the one hand, the ones with lung or brain metastases, and on the other, the ones with intraperitoneal spread resembling advanced ovarian cancer. In the current FIGO 2023 staging, substages IVB and IVC were distinguished, where IVB refers to the patients with peritoneal carcinomatosis above the pelvis and stage IVC refers to distant metastasis including metastasis to any extra- or intra-abdominal lymph nodes above the renal vessels, lungs, liver, brain, or bone [3]. These changes were made as it was noticed that treatment decisions vary significantly in cases with limited pelvic versus extrapelvic peritoneal carcinomatosis [3,26]. Patients with only intraabdominal disease are candidates for radical surgery that often resembles cytoreductive surgery for advanced ovarian cancer. In patients with extra-abdominal disease, radical pelvic/abdominal surgery with metastasectomy is possible only for a selected group with oligometastatic disease [45].

Peritoneal carcinomatosis (IVB stage according to FIGO 2023) is generally rare in EC (approximately 2%), but these patients should be distinguished from those with distant metastases, as in this subgroup, maximal/optimal cytoreduction is more feasible [45]. There is much evidence that the extent of cytoreduction correlated with survival, with the maximum benefit if no macroscopic disease remained [39,46,47]. In the cohort of 929 patients, it was noticed that peritoneal metastases were associated with significantly better OS than organ-specific metastasis (median OS: 29 vs. 19 months, respectively), while bone, brain, and lung metastases were identified as independent prognostic factors for worse OS [45]. In another study, higher performance status, age below 59 years, and adjuvant chemotherapy followed by radiotherapy were significantly associated with superior survival [39].

In a study by Ueda et al. on 30 EC patients, optimal cytoreduction (defined as residual disease ≤2 cm) was associated with improved PFS and OS, not only among the patients with exclusively intra-abdominal metastasis but also among cases with extraabdominal disease, providing evidence that aggressive cytoreductive surgery for stage IVC (FIGO 2023) EC should also be considered [48]. Nevertheless, surgery can be beneficial even in patients without chances for complete resection. A study by Guo et al. analyzed data of 730 EC patients with at least one extra-peritoneal metastasis to either the lungs, bones, or brain. Approximately half of the patients underwent palliative pelvic surgery but without resection of the metastases. A significant benefit in median survival was observed (9 vs. 23 months for lung, 8 vs. 19 months for bone, and 4 vs. 15 months for multiple organ metastases, respectively) for the operated group, except for the patients with brain metastasis [49]. Regarding lymphadenectomy, routine systemic pelvic and paraaortic lymphadenectomy in advanced EC is not recommended in the absence of suspicious lymph nodes on pre-operative imaging and intraoperatively and should only be performed as part of cytoreductive surgery (see more in the chapter on lymphadenectomy) [18].

The most important findings on surgical treatment of early and advanced EC are summarized in Table 1.

## 5. Treatment of Primary Unresectable Disease

If preoperative assessment indicates no possibility for optimal cytoreductive surgery, neoadjuvant chemotherapy followed by surgery or definitive radiotherapy can be offered. Although complete radiologic response to neoadjuvant chemotherapy in EC is low (approximately 3–4%), partial response is achieved in as many as 72–76% of the patients, facilitating interval debulking surgery, which was attempted in 76–78% of the patients. Complete macroscopic resection was possible in 52–60% of the patients, and optimal debulking in a further 29% of the operated patients. Interval debulking surgery after chemotherapy provided a significant PFS and OS benefit compared to not operated patients (11.53 vs. 4.99 months for PFS and 24.13 vs. 7.04 months for OS), independently of the histological type of EC [53,54]. In a cohort study on 4890 patients, two approaches to treat stage IV EC were compared: neoadjuvant chemotherapy followed by interval debulking surgery and primary debulking surgery followed by adjuvant chemotherapy. The study found a survival benefit for the neoadjuvant chemotherapy group in the first 3–8 months after initiation of therapy but better survival in the primary surgery group in the long run [55]. Another small study reports similar survival outcomes for both groups, with the benefit of decreased operative time and hospital stay in the neoadjuvant chemotherapy cohort [56].

As mentioned above, there is also some evidence showing that neoadjuvant radiotherapy enables subsequent optimal surgery in locally advanced EC [57,58]. However, radiation significantly hampers surgery; therefore, in the case of patients that cannot be treated with primary surgery, neoadjuvant chemotherapy and radiation therapy following the surgical management can be preferred.

To conclude, neoadjuvant chemotherapy followed by surgery is a treatment option for primary unresectable disease as well as for some patients with locally advanced disease, where the shrinkage of tumors due to chemotherapy enables less extensive surgery and avoidance of stoma (see Table 1).

## 6. Palliative Surgery

Occasionally, primary advanced or recurrent EC is associated with troublesome and/or life-threatening symptoms such as vaginal bleeding or vaginal fistulas. In some cases, pelvic exenteration can be offered as palliative treatment if the patient’s quality of life is immensely affected by symptoms such as tumor necrosis, fistulae, and severe pain, providing a relief. However, each time, the surgical morbidity and potential benefits have to be carefully balanced. Palliative simple hysterectomy can sometimes be offered in cases of uncontrolled uterine bleeding and advanced/metastatic disease when radiotherapy is not an option. Data from two retrospective SEER analyses suggest that apart from symptom control, palliative hysterectomy is likely to be associated with a survival benefit. The paper by Guo et al. analyzed the impact of non-radical surgery in EC with extraperitoneal metastases and found that surgery was associated with a significant benefit in cancer-specific survival in patients with pulmonary, bone, and multiple organ metastases but not for patients with brain metastasis [49]. Another SEER analysis on 4072 patients with stage IVB EC that underwent cancer-directed surgery showed a survival benefit in all groups, including brain metastasis [59].

## 7. Lymph Node Dissection in EC

For many years, lymphadenectomy in EC patients has been a controversial issue. Currently it seems that many controversies were solved with the advance on sentinel lymph node biopsy (SLNB).

The extent of the lymph node resection in EC may vary according to the situation. It may be limited to SLNB only, it may have the form of selective lymphadenectomy of the suspicious lymph nodes identified either by palpation or radiologically, or full systematic pelvic and/or paraaortic lymphadenectomy may be performed. Lymphadenectomy in EC is a complex issue: on the one hand, relatively few patients have metastatic lymph nodes, the procedure is associated with increased surgical risk, and there is no strong evidence that systemic lymphadenectomy improves survival. On the other hand, lymph node status is important for risk stratification and thus optimal planning of the adjuvant treatment and removal of the metastatic lymph nodes has prognostic significance. Herein, the pros and cons of different forms of lymphadenectomy and supporting scientific evidence are summarized.

The reported overall rate of positive lymph nodes in EC was 5–9% in the entire cohort and 20–29% in the high-risk group [51,52]. Among the patients with positive lymph nodes, about a half had both pelvic and para-aortic involvement [60]. Isolated pelvic metastases were reported in around 35% of the patients. The rate of isolated para-aortic metastases ranged from 1–3% [60,61,62] to as much as 15–16% [63,64]. Furthermore, a majority (67–77%) of patients with para-aortic lymph node involvement had metastases above the inferior mesenteric artery [60,63], indicating that systematic lymphadenectomy in EC—if indicated—should involve both pelvic and para-aortic regions up to the level of the renal veins. Pelvic lymphadenectomy should extend from the mid-portion of the common iliac vessels superiorly to the circumflex iliac vein inferiorly and from the mid-psoas muscle laterally to the ureters medially, including the obturator fossa [65].

The presence of lymph node metastases has a significant negative impact on prognosis—the five-year survival rates for patients with vs. without lymph node involvement oscillate around 61% vs. 90%, respectively [66]. Several factors were identified to influence the risk of lymph node metastases in EC and should be taken into account when qualifying the patients for lymph-node status assessment: tumor stage and grade; histological type; lymphovascular space invasion (LVSI); depth of myometrial infiltration (none/less than 50%/more than or equal to 50%); tumor size; and, most recently, molecular type of EC [67,68,69,70,71] (see Table 2).

## 8. Lymph Node Staging in Early EC (FIGO I-II, T1-2)

There are no clear data showing that systematic pelvic lymphadenectomy in early stage EC improves DFS or OS. The ASTEC multicenter trial on 1408 patients with EC confined to the uterine corpus (staging based on the preoperative assessment) showed no benefit in terms of OS and RFS for routine pelvic lymphadenectomy [75]. Another randomized controlled trial on 514 patients showed no benefit in DFS and OS; however, it underlined that it did improve surgical staging—significantly more patients with lymph node metastases were found in the lymphadenectomy group than in the no-lymphadenectomy group (13.3% vs. 3.2%) [76]. Naturally, systematic lymphadenectomy was associated with more post-operative complications, although adverse events were generally rare and not severe. In both studies, the patients without lymphadenectomy were more likely to receive adjuvant radiotherapy. Thus, one of the potential benefits of surgically confirmed negative lymph node status would be avoidance of unnecessary post-operative radiotherapy. One of the major limitations of the above-mentioned trials is performing only pelvic lymphadenectomy and lack of para-aortic lymphadenectomy. As mentioned above, about a half of patients with metastases in the pelvic lymph nodes also had metastases in the para-aortic lymph nodes, and on top of that, up to 16% had isolated para-aortic metastases [63,64]. These statistics bring into question the curative impact of lymphadenectomy limited to the pelvis. The fact that in the majority of cases, radical radiotherapy cannot be applied to the bulky metastases in the paraaortic region is another argument for performing full pelvic and paraaortic lymphadenectomy in EC.

On the other hand, meta-analyses of observational trials and retrospective analyses suggest better survival of EC patients after lymphadenectomy. In a retrospective cohort study on 151,089 women with early stage EC, performance of any lymphadenectomy was associated with a 16% reduction in mortality [77]. Reduction in mortality was observed for stages FIGO IB and FIGO II but not for FIGO IA. Nevertheless, it is hard to draw certain conclusions from these data as the extent of lymphadenectomy varied significantly between the patients, and a subgroup analysis suggested no association between extensive lymphadenectomy (number of nodes removed <10 vs. ≥10) and survival for patients with FIGO stage I EC [77]. A 2017 Cochrane review found no evidence that lymphadenectomy decreases the risk of death or disease recurrence compared with no lymphadenectomy in women with presumed FIGO stage I disease and underlined the surgical risks related to lymphadenectomy as well as long-term complications (lymphoedema/lymphocyst formation) [78]. On the contrary, in the matched cohort analysis limited to stage I endometrioid EC, pelvic lymphadenectomy was associated with increased survival compared with no lymphadenectomy (five-year survival 91.4% vs. 87.3%). Furthermore, the addition of para-aortic lymphadenectomy was associated with increased survival compared with pelvic lymphadenectomy alone (five-year survival 91.0% vs. 89.8%) [79]. Two meta-analyses based on retrospective observational studies encompassing 8 [80] and 13 studies [81] (some overlapping) report a survival benefit of combined pelvic and paraaortic lymphadenectomy for intermediate and high-risk EC patients. One revealed a stunning 46% decrease in mortality, 13% increase in five-year OS rate, and 23% increase in five-year DFS rate for combined pelvic and para-aortic lymphadenectomy compared with pelvic lymphadenectomy only in patients with intermediate/high-risk EC [81]. What is worth underlining is that the survival benefit was not observed for patients with low-risk disease [82]. Nevertheless, caution needs to be taken when interpreting these results as some analyses revealed that the no-lymphadenectomy group had more risk of death due to cardiovascular and pulmonary diseases, suggesting a bias in that only medically fit patients were qualified for this procedure [83,84].

There are some data suggesting a correlation of the number of the removed nodes with survival. In patients with grade 3 EC, removal of more than 11 pelvic lymph nodes was associated with improved OS and PFS compared with removal of less than 11 pelvic lymph nodes. This association was not observed for grade 1 and 2 EC [84,85]. This correlation might be explained by the cytoreductive potential of lymphadenectomy or a bigger chance of finding small/isolated nodal metastases with the increasing number of nodes removed. However, the survival benefit could also be attributable to the fact that in the clinical setting, a more radical lymphadenectomy is more often performed in younger, thinner, and generally healthier patients. The international guidelines do not state the minimum number of lymph nodes to be removed in order to confirm negative lymph node status of a patient [18,19].

Correct staging and qualification for adjuvant treatment is one of the strongest arguments for assessing lymph node status in EC patients. Firstly, positive lymph nodes qualify for adjuvant systemic treatment with chemotherapy. Secondly, surgically confirmed negative lymph nodes help to avoid overtreatment and morbidity related to unnecessary adjuvant treatment. A negative correlation between the rate of lymphadenectomy and adjuvant radiotherapy was observed [86].

In order to find a compromise between proper staging that affects adjuvant treatment decisions and acceptable morbidity, the procedure of sentinel lymph node biopsy (SNLB) was introduced. The negative predictive value of the SLNB procedure in EC proved to be excellent and reaches 99% [87,88,89]. Surprisingly, the SLNB appeared to also be more sensitive than standard evaluation after systematic lymphadenectomy. Numerous studies confirmed the superiority of the SLNB over systematic lymphadenectomy to detect positive pelvic nodes: the reported rate of patients with positive pelvic lymph nodes detected by SLNB are approximately twofold greater than by systematic lymphadenectomy, with a lower median number of lymph nodes removed [90,91,92]. No difference in the detection of positive paraaortic nodes was observed [92]. Interestingly, it was observed that the sentinel node was the only metastatic node in as much as 50% of the patients, and micrometastases or isolated tumor cells were common (62.9%) [93].

High sensitivity of the SLNB with the indocyanine green (ICG) procedure for determining lymph node status was shown in a meta-analysis from 2019 [92], and the introduction of ultrastaging further improved its sensitivity for detection of small metastases [94,95]. In the cohort of patients with grade 1 and 2 endometrioid lesions with myometrial invasion less or equal to 50% (FIGO stage IA) and a tumor diameter less or equal to 2 cm, there was 0% of lymph node involvement using a standard technique [96]. However, in another study on 425 patients with grade 1–2 EC and myometrial invasion less or equal to 50%, the reported rate of positive lymph nodes was 5.9%, almost half of which were detected only after ultrastaging [94]. The FIRES trial revealed excellent sentinel lymph node accuracy in detecting metastases for EC FIGO stage I of all grades and histopathological subtypes [97] and the SHREC trial specifically for high-risk EC [98]. More evidence emerged that SNLB can be a safe alternative to systematic lymphadenectomy in the intermediate–high- and high-risk EC groups [99]. However, it is argued that in the high-risk EC, the SLNB has limited significance as this group is qualified for adjuvant chemotherapy independently of the lymph node status [18,19]. It is estimated that SLNB can influence the decision on the adjuvant treatment in 7.9% of patients in presumed early stage low-risk EC [100]. In a nation-wide retrospective analysis of nearly 900 patients with low-risk EC, as much as 12.1% were found to have nodal involvement after SLNB (22% macrometastases and 50% micrometastases), underlining the importance of SLNB in the surgical management of patients with early stage EC [101].

SNLB can be safely performed if macroscopic disease had been ruled out by adequate imaging. In patients with SNLB only, despite nodal metastases, the prognosis was not inferior to those after systematic lymphadenectomy [92,102,103]. Finally, SNLB provides a chance for verifying lymph node status in patients in whom systematic lymphadenectomy cannot be performed due to general medical conditions. The SLNB approach provides reduced duration of the surgery, limits peri-operative morbidity and long-term complications, and reduces the time of hospitalization [104], having thus currently emerged as a recommended approach to apparent early stage EC.

## 9. Lymphadenectomy in EC Patients with Lymph Node Metastases

The available data from retrospective studies indicate that the removal of bulky lymph nodes improves survival and should be treated as part of the cytoreductive surgery [36,38,105,106]. For the detection of lymph node metastases in EC, standard imaging methods such as CT scan, MRI, and PET-CT are characterized by low sensitivity (around 50%) but high specificity (around 95%) [107]; therefore, if suspicious lymph nodes are detected by preoperative imaging, macrometastases are highly likely, and removal of these lymph nodes is part of cytoreductive surgery and should always be performed. Intraoperative inspection or palpation of nodes should not be used to detect metastatic lymph nodes, as less than 10% of patients with positive lymph nodes had grossly enlarged nodes [71]. As mentioned in the chapter on surgical management of advanced EC, a large meta-analysis indicated that suboptimal (≥1 cm vs. <1 cm) cytoreduction was associated with worse PFS and OS [36]. Another study specifically on a subgroup of 41 patients with FIGO stage IIIC EC identified complete resection of macroscopic nodal disease and the administration of adjuvant chemotherapy (in addition to radiation therapy) as independent predictors of disease-specific survival [106]. Median disease-specific survival for patients with complete resection of the macroscopic lymphadenopathy differed significantly compared to patients with gross residual nodal disease (37.5 vs. 8.8 months, respectively) [106]. A similar study provides concordant data estimating a five-year disease specific survival at 63% for completely resected nodal disease versus 43% for those with macroscopic residual disease [108].

Although no clinical trials are available, studies that investigated the role of para-aortic lymphadenectomy in addition to pelvic lymphadenectomy in patients with stage III EC suggest its therapeutic role [105,109]. It is worth emphasizing that the studies cited above refer to grossly enlarged lymph nodes. The data on the therapeutic role of systematic lymphadenectomy in the case of nodal micrometastases are unclear. In a retrospective analysis on 104 stage IIIC EC patients with only microscopic nodal metastases (>0.2 mm), the authors found no significant benefit in survival for the group undergoing systematic pelvic and para-aortic lymphadenectomy compared with the sentinel lymph node algorithm with limited nodal dissection [110]. This effect might be explained by the fact that adjuvant radio-/chemotherapy is effective against microscopic disease but less effective against macroscopic (>2 cm) disease. Especially radiotherapy on the para-aortic region has its limitations due to the proximity of the small intestine [111]. In our institution, in the case of lymph node metastases, we perform systematic rather than selective lymphadenectomy. The remaining lymph nodes may be the source of recurrence, despite adjuvant chemotherapy and radiotherapy.

In summary, in apparent early stage EC, systematic lymphadenectomy can be replaced by SLNB with ICG. Bulky lymph nodes should be removed as part of the cytoreductive surgery in advanced EC. However, in the case of metastases to the lymph nodes, we prefer systematic lymphadenectomy of the pelvic and paraaortic region up to the level of the renal vessels. The evidence on lymphadenectomy in EC is summarized in Table 3.

## 10. Oophorectomy in EC

Removing the ovaries in EC has two main goals: removal of the micrometastases and reducing the estrogen levels that might promote endometrial proliferation. The detrimental effects of estrogen deprivation mainly affect the population of women younger than 45 years. In this group, premenopausal oophorectomy without hormonal replacement therapy was associated with a dramatic 44–67% increase in the mortality [112,113]. While in early EC, ovarian metastases are exceptional, in high-grade endometrioid or non-endometrioid histology, occult ovarian metastases were observed in 2–3% of the patients [114]. In premenopausal women with early stage EC, ovarian preservation seems not only safe [115,116] but in some reports is even associated with decreased mortality due to cardiovascular events in low-risk patients [117]. A meta-analysis from 2017 showed no significant difference in OS between the patients that underwent bilateral salpingooophorectomy and those who preserved their ovaries [52]. Similarly, in a large multicenter study, ovarian conservation did not negatively impact OS in early stage and low-grade EC [118], nor the rate of recurrence [115,117]. Nevertheless, nowadays, it is still unclear whether and when ovarian preservation can be safely offered to premenopausal women with low-risk EC. However, when low-risk EC is recognized after surgery for benign conditions, then the ovaries may be preserved.

## 11. Surgical Management of Non-Endometrioid EC

Non-endometrioid ECs are rare, and therefore the evidence from the studies is usually limited. Similarly to endometrioid EC, laparoscopy was not inferior to laparotomy for the surgical treatment of high-risk EC, as indicated by a meta-analysis including 2332 patients [119]. Recently, more evidence has emerged that SNLB can be a safe alternative to systematic lymphadenectomy in the intermediate–high- and high-risk EC groups [99]. The use of the SLNB procedure was associated with a similar OS as systematic pelvic and paraaortic lymphadenectomy in serous and clear cell carcinoma [102].

Serous EC appears to have some similarities with serous high-grade ovarian cancer. One of the features that is different for this subtype of EC is the prognostic significance of positive pelvic cytology [120,121], and therefore in this group, pelvic washings or pelvic biopsy seems reasonable despite not being reflected in the FIGO classification. Furthermore, a higher prevalence of BRCA mutations was observed in patients with serous EC [122], which might justify screening for BRCA1/2 mutations in this group. Another common feature with serous ovarian cancer is the high rate of omental metastases. For serous EC, around 17–19% of patients are found to have omental metastases, half of which are microscopic [50,51], making omentectomy a recommended staging/cytoreductive procedure in serous EC [18]. For advanced serous EC, the radicality of the cytoreductive surgery is the most impactful on survival. The optimal cytoreduction (<1 cm of residual disease) was associated with a significantly better OS than suboptimal debulking (>1 cm)—the estimated benefit in the median survival was 24 vs. 9.6 months [40] and 52 vs. 16 months [123].

Clear cell EC is an aggressive subtype characterized by a significant proportion of positive LVSI (41–58%) [124,125], omental metastases in 12.5% and nodal metastases in 16.3% [124]. In clear cell EC, a trend towards impaired RFS but with no difference in OS was observed in the SLNB group [102]. Similarly to serous EC, the peritoneal cytology has a prognostic value and indicated a higher recurrence and inferior survival [126]. According to the data on the molecular types of EC, a significant proportion (35%) of clear cell ECs are characterized by p53 abnormality [127]. The surgical approach in early stage disease involves hysterectomy, with lymphadenectomy recommended [18,19]. For clear cell EC, a trend towards impaired RFS but with no difference in OS was observed for the SLNB procedure [102]. In advanced disease, maximal cytoreduction is desired [36].

Carcinosarcoma is the most aggressive EC subtype, therefore requiring the most extensive surgical treatment. Omental metastases are found in 16% of the patients [128], and although omentectomy did not alter survival, it had a strong prognostic significance. The surgical approach to apparent stage I carcinosarcoma should involve staging infracolic omentectomy [18]. In the case of a more advanced disease, a radical hysterectomy or even pelvic exenteration might be indicated [19]. The SLNB seems to be a safe approach [129], although many studies suggest a curative role of a full staging surgery (including pelvic lymphadenectomy). A retrospective analysis on 1140 patients concluded that lymphadenectomy (defined as removal of any lymph nodes) improved survival when more than 10 nodes were removed [130]. In a matched cohort study of 5614 patients with stage I carcinosarcoma, omitting lymphadenectomy was associated with decreased median survival (45.2 vs. 73.9 months), and the removal of at least 15–20 lymph nodes was associated with increased survival [131]. Whether paraaortic lymphadenectomy improves survival remains unclear [132].

## 12. Surgical Treatment of Recurrent Disease

The treatment of recurrent EC depends on the type and location of the recurrent disease (see Table 4), on the prior treatment, and on the general condition of the patient. This review focuses on the role of surgery in recurrent EC. The clinical presentation of recurrent EC may have various patterns (local, regional, nodal, peritoneal, or distant) and therefore different treatments. Unfortunately, many papers do not report precisely the type of recurrence, and instead, all types of recurrent disease are reported jointly. In order to better define EC recurrence risk groups and develop specific guidelines for each type, a classification for recurrent EC was proposed by the French group FRANCOGYN. After analyzing data of 1230 women with EC initially treated by primary surgery, a recurrence rate of 18.2% was observed, and four major dissemination pathways were identified: locoregional, lymphatic, hematogenous (distant organ recurrence), and peritoneal. The majority of the patients (75.8%) had a single-pathway recurrence, and among those women, significant differences in the five-year OS and survival after recurrence were observed, with the worst prognosis for the peritoneal carcinomatosis pattern of spread [133].

The locoregional recurrence can be subdivided into two groups with different prognoses: vaginal cuff recurrence and pelvic recurrence, where the latter is usually associated with bladder or rectal infiltration. A locoregional recurrence is rather uncommon (7.1% of the recurrences occur in the vaginal cuff and 8.1% in the pelvis) and occurs more often in patients without previous radiation treatment [134]. For previously non-irradiated patients with isolated locoregional recurrence, radiotherapy is the method of choice. In some cases, surgical removal of the easily accessible tumors can be considered for better local symptom control prior to radiotherapy [18]. However, it was shown that the effect of radiotherapy depended on the tumor size and was significantly worse for tumors >2 cm [135], suggesting that this group of patients might especially benefit from surgical resection. In another study on vaginal vault recurrent EC in previously non-irradiated patients, the advantage of surgical treatment over radiotherapy was observed with re-recurrence rates within 2 years of 40% vs. 0% for radiotherapy and surgery, respectively, as well as a benefit in two-year survival rates (83% vs. 100%) [136]. However, it should be noted that from 33 patients in the study, only 5 were qualified for surgical treatment only, which emphasizes the fact that only proper qualification for surgery can provide a substantial survival benefit. Although there is some evidence that surgical treatment of vaginal vault EC recurrences in previously non-irradiated patients provides a benefit over radiotherapy [136], the European guidelines still recommend radiotherapy with or without chemotherapy as the primary method for locoregional recurrent EC in previously non-irradiated patients [18].

Locoregional EC recurrence in patients that were already treated with external beam radiation to the pelvic area is usually an indication for pelvic exenteration. In this group, repeating radiotherapy can also be considered, but its high morbidity, mainly the formation of fistulas, has to be taken into account [134]. If neither radiotherapy nor surgery is possible, palliative systemic treatment with chemotherapy and/or immunotherapy can be offered [18].

Pelvic exenteration involves resection of the uterus or the vaginal cuff and other pelvic organs, and it can be divided into anterior (where the urinary bladder is resected), posterior (where the rectosigmoid is resected), total (where both the urinary bladder and the rectosigmoid colon are resected), or modified with modifications as to the extent of the organ resection. Considering the depth of resection, exenteration can be classified into type I (supralevator), type II (infralevator), and type III (with vulvectomy) [137]. Naturally, the mode of resection depends on the location, size, and consequences (e.g., fistulas) of the recurrent disease. Most commonly, recurrent EC is associated with infiltration of the trigone of the urinary bladder anteriorly +/− infiltration of the ureters and with infiltration of the anterior wall of the rectum at the level of the rectouterine pouch posteriorly; therefore, total supralevator pelvic exenteration is mostly performed [44]. In the rare case of tumors extending to or fixed to the pelvic side wall, a technique called laterally extended endopelvic resection (LEER) can be proposed [138]. LEER is based on the resection of the internal iliac vessel system and encompasses, depending on the tumor size and location, total resection of the mesorectum, mesometrium, and mesovesical space with pelvic side wall and floor muscle (e.g., obturator internus, pubococcygeus, iliococcygeus, and coccygeus muscles) excision. Infiltration of the lumbosacral trunk and the sacral plexus located dorsally and laterally to theobturator internus muscle are considered irresectable. Although associated with substantial morbidity (moderate to severe treatment-related morbidity was reported in as much as 70% of the patients), LEER can be offered to selected patients as salvage treatment with a curative aim, with a reported five-year recurrence-free probability of 62% [138].

Pelvic exenteration is a procedure associated with significant rate of early post-operative complications and a decreased quality of life, mainly due to stomas (colostomy, ileostomy, urostomy). It carries a 3% risk of perioperative death and a further 21–22% risk of major post-operative complications [139,140]. Thereby, it should only be offered if complete resection with clear surgical margins seems feasible and after the spread of metastatic disease had been ruled out on imaging, preferably PET, which has the highest positive predictive value (approximately 92–94%) [141]. To justify high surgical burden, the primary aim of such surgery should be curative rather than palliative. Additionally, histopathological confirmation of the recurrent disease is highly recommended to avoid a mistakenly diagnosed recurrence, which can happen in as much as 8.6% of the cases [142]. Completeness of cytoreduction is the main factor affecting long-term survival underlining the role of proper patient qualification for treatment—the reported three-year survival among patients who had pelvic exenteration with no residual disease was 48%, while for the 1–2 cm residual disease group, it dropped to 22% [143]. Other identified positive prognostic factors were lack of metastatic lymph nodes and negative LVSI [143,144]. The presence of pelvic sidewall involvement and/or hydronephrosis did not negatively affect survival [145]. The reported five-year survival rates for patients undergoing pelvic exenteration due to recurrent endometrial cancer range from 40% to even 80% [139,144,146], showing that it is a valuable treatment option for a selected group of patients, offering a substantial prognostic benefit.

Abdominal recurrences are the most common pattern of recurrent EC. Cytoreductive surgery for recurrent EC, similar to that applied to advanced ovarian cancer, has recently been intensively investigated. It is defined as the removal of all macroscopic disease, including single site and locoregional recurrences, and therefore the results presented in this section might partially overlap with the evidence cited above. Contrary to pelvic exenteration, cytoreductive surgery may extend beyond the pelvis and therefore can be considered a treatment option for patients with abdominal spread that were previously considered inoperable. A recent systematic review on this topic, gathering evidence from 11 retrospective studies comprising 1146 patients, concludes that cytoreduction (in combination with other methods) in recurrent endometrial cancer is associated with prolonged OS and PFS [147,148,149,150]. Similarly to ovarian cancer, completeness of resection was the most important prognostic factor, and the size of residual disease directly correlated with survival, with the reported median OS ranging from 39 to 76 months for complete debulking (no macroscopic disease) [148,151,152] and from 9 to 22 months for suboptimal debulking (>1 cm of residual disease) [151,153]. Complete cytoreduction was obtained in the majority of the patients (57–75%) [147]. Importantly, for proper patient selection, factors that positively correlated with achieving complete cytoreduction were identified: solitary disease, tumor size <6 cm, and ECOG performance status of 0 [147,151]. Other identified positive prognostic factors after secondary cytoreductive surgery were the lack of metastatic lymph nodes, negative LVSI, and endometrioid histology [72]. Interestingly, no correlation was observed between previous radiotherapy and achieving complete cytoreduction [147]. In conclusion, the available evidence from retrospective studies indicates that in recurrent EC, complete cytoreductive surgery combined with other methods (radiotherapy; chemotherapy; hormonal therapy; and, most recently, immunotherapy) provides the largest survival benefit. However, careful patient selection for secondary debulking surgery is crucial to optimize the results.

Extra-abdominal EC recurrences are observed in as many as 32.8% of the patients [133], with the majority of distant metastases found in the lungs [49]. In most cases, systemic therapy is offered. Nevertheless, in the case of isolated disease, there are limited data on the role of surgical management. A review by Tangjitgamol et al. assessed the role of surgery to treat isolated pulmonary, hepatic, and cerebral metastasis, showing a potential benefit in selected patients who had good performance status, long disease-free interval, absence of other systemic diseases, and clear margins [154].

Isolated lymph node recurrence occurs rarely (see: Table 1) and can be treated with either surgery, radiotherapy, or chemotherapy. If complete resection is possible with acceptable morbidity, surgery should be the method of choice [18]. Similarly, in the case of oligometastatic disease (typically defined as 1–5 metastases), radical therapy, including surgical removal, radiotherapy, or local ablation techniques, should be considered, as there is growing evidence suggesting a long-term survival benefit [155,156]. In the case of multimodal recurrence type, individual counselling is needed, and palliative systemic treatment is usually offered.

In summary, for treatment of recurrent EC, radical surgery should be considered if complete resection with clear margins is deemed possible. Surgical techniques can range from local excision of a tumor of the vaginal vault through to pelvic exenteration to extensive secondary cytoreductive surgery. For patients who underwent complete surgical resection, a substantial survival benefit is noted.

## 13. Future Directions

One of the future directions that may alter the scene for EC surgery is the impact of molecular profiling for choosing the most appropriate surgical approach. It was observed that EC molecular subtype strongly correlated with the presence of lymph node metastases (see Table 5) [157,158]. In the p53 mutation subgroup, associated with worse prognosis, lymph node metastases were observed in as much as 38–45% of the patients [157,158].

One of the main concerns related to systemic lymphadenectomy in EC is that the evaluation of conventional risk factors (histopathological type, tumor grade, depth of invasion) and imaging studies failed to identify a group of patients at very high risk of lymph node metastases.

The analysis of the p53 mutation may help distinguish this group of patients. However, despite the high incidence of lymph node metastases in p53-mutated ECs, it is not clear whether these patients would benefit from systematic lymphadenectomy. Firstly, p53 mutated ECs are considered high-risk tumors, and therefore adjuvant chemotherapy is recommended independently of the lymph node status. Secondly, as stated before, there is no clear evidence that systematic lymphadenectomy in the case of macroscopically unchanged lymph nodes improves survival in EC. This observation may be especially relevant in the case of p53-mutated ECs, where most recurrences are outside of the lymphatic system. Aznar et al. analyzed the recurrence pattern according to the molecular profile of EC. Most p-53 mutated patients had either distant site or peritoneal recurrence, while nodal-site recurrence was found only in 14.7% of patients [162].

Similarly, Jamieson et al. investigated long-term outcomes of stage I p53-abnormal low-grade endometrioid Ecs; the predominant type of recurrence was distant recurrence [163]. Therefore, despite the high rate of lymph node metastases in p53-mutated Ecs, the exact impact of lymphadenectomy on long-term outcomes is hard to predict, and prospective clinical trials are needed to provide evidence on this issue. Nevertheless, p53-mutated EC is classified as high-risk disease, and therefore surgical evaluation (at least SLNB) of the lymph nodes is currently considered essential.

There is conflicting data regarding the possibility of deescalating surgical treatment of POLEmut EC patients. The rate of the lymph node metastases in this group significantly differs between the studies (see Table 5). Some reported a very low incidence of lymph node metastases in POLEmut EC and speculated that lymphadenectomy or even SLNB in this group may be abandoned [158]. In the study by Vrede et al., only 3% of POLEmut ECs had positive lymph node [159]. However, in the other studies, the authors reported a much higher (8.7–14.2%) rate of lymph node metastases in the POLEmut EC patients [157,161]. McAlpine et al., in the meta-analysis including 359 POLE-mutated ECs, showed lymph node metastases in 5.2% of patients [160]. Interestingly, no isolated paraaortic lymph node metastases were found in the POLE cohort [157]. These results suggest that lymph node metastases are less frequent in POLEmut ECs than in the other molecular subgroups; however, their incidence is still clinically relevant.

Furthermore, early stage POLEmut ECs without lymph node metastases have excellent prognosis, reaching 98% of 10-year cancer-specific survival [164]. The exact prognosis of POLEmut cancers with lymph node metastases is unknown. POLEmut ECs constitute a relatively small group (<10%) of EC; therefore, the data concerning prognosis and management of advanced-stage patients is limited. We think that it is too early to deescalate surgical management of this group of patients. The excellent quality data obtained from prospective trials are essential to change the current management.

Interesting results were found in the study by Bilir et al., which analyzed the MMRd/MSI subgroup. The authors observed differences in lymph node metastases according to defects in particular elements of the MMR system. The authors showed that abnormal staining of MSH2 and MSH6 did not correlate with lymph node metastasis; however, all lymph node metastatic patients showed PMS2 and MLH1 [158]. If these results are confirmed in the prospective trials, another relevant risk factor for lymph node metastases will be obtained.

The remaining challenge is that the molecular subtype is currently best diagnosed on a post-surgery specimen. Similarly to the grade assessment, a significant discordance in the detection of the p53 abnormality was observed between preoperative biopsy specimens and hysterectomy specimens. It is reported that preoperative biopsy failed to exhibit abnormal p53 staining in 9% of the tumors, while the overall discordance between the pre- and post-operative evaluation was exceeding 16% [165].

In summary, although molecular profiling of EC adds important information concerning disease-related risk, the data are insufficient to change the current surgical management and mostly impact the adjuvant therapy of EC.

Finally, thanks to the molecular profiling of EC, new adjuvant therapies (e.g., immunotherapy) were introduced. The introduction of immunotherapy significantly altered the management of EC, especially in the MMRd cohort. Future trials concerning EC will have to consider these new strategies in adjuvant therapy and their impact on the DFS and OS, and therefore the role of surgery may also change.

## 14. Summary

The aim of this narrative review paper was to summarize the available literature on the surgical management of EC. Surgery still plays a key role in the treatment of EC. However, due to the increasing knowledge on the pathology and molecular features of EC, as well as the new advances in the adjuvant therapies, the surgical management of EC has become more complex. Most of the available research grouped EC according to the histopathological type, whereas the application of the molecular classification could completely shuffle these groups and immensely alter the results of EC subgroup analysis. More research in the field is to be expected. Meanwhile, the modern approach to EC treatment makes it essential to adjust the extent of surgery to a particular patient, ensuring an optimal, made-to-measure personalized surgery.

## Figures and Tables

**Table 1:** Range of resection according to EC stage—summary.

Stage (FIGO 2009/TNM)	Surgical Procedure	Results	Type of Study	References
FIGO I/T1a-1b	Hysterectomy	Five-year DFS and OS were similar for modified radical hysterectomy and simple total extrafascial hysterectomy (DFS of 87.7 and 88.9% and OS of 89.7 and 92.2%, respectively)	Randomized controlled trial	[13]
	Vaginal cuff resection	Conflicting data: No correlation between the recurrence rate and the length of vaginal cuff resection	Retrospective	[20]
		Transection of vaginal cuff is an independent prognostic factor in stage I endometrial cancer (five-year DFS was 91.8% for patients with vaginal cuff resection compared with 83.7% for patients without vaginal cuff)	Retrospective	[21]
	Omentectomy	Yes in serous and undifferentiated EC (rate of omental metastases 17–19%)	Retrospective	[50,51]
		No for endometrioid EC (rate of omental metastases 3%)	Meta-analysis	[23]
	Oophorectomy	No difference in OS with/without oophorectomy in early stage EC	Systematic review and meta-analysis	[52]
FIGO II/T2	Hysterectomy	Similar DFS and OS for radical hysterectomy vs. simple hysterectomy	Meta-analysis	[30]
FIGO III-IVA	Maximal cytoreduction	Leaving any gross residual disease is associated with worse PFS rates (HR, 2.16) and worse OS rates (HR, 2.57)	Systematic review and meta-analysis	[36]
FIGO IVB (2023)	Maximal cytoreduction	The extent of cytoreduction correlated with survival, with the maximum benefit if no macroscopic disease remained. The median survival for optimal surgery was 34.3 months compared to 11.0 months for >1 cm residual disease	Retrospective	[47,39]
		In the optimal cytoreduction group (no macroscopic disease), the median survival was 48 months compared to 13 months in patients with remaining macroscopic disease	Retrospective	[47]
FIGO IVC (2023)	Maximal cytoreduction	Optimal cytoreduction (residual disease ≤2 cm) was associated with improved PFS and OS in patients with extraabdominal disease	Retrospective	[48]
Primary unresectable disease	Interval debulking surgery after neoadjuvant treatment	Median OS was 41 months after complete (no gross residual disease) and optimal (<1 cm gross residual disease) debulking, 16 months after incomplete debulking, and 13 months for patients who did not undergo surgery	Retrospective	[53]

**Table 2:** The risk of lymph node metastases in EC according to stage and grade [72,73,74].

Depth of Myometrial Invasion	Grade		
	G1	G2	G3
<50%	0–4%	3–16%	5–15%
≥50%	0–9%	14–20%	17–28%

**Table 3:** Lymph node resection in EC.

Stage (FIGO 2009/TNM)	Results	Type of Study	References
**FIGO I/T1a-1b**	No benefit in terms of OS and RFS for routine systematic pelvic lymphadenectomy	Randomized controlled trial	[75]
	No benefit in terms of OS and DFS for routine systematic pelvic lymphadenectomy	Randomized controlled trial	[76]
	Performing any lymphadenectomy was associated with a 16% reduction in mortality for stages FIGO IB and FIGO II (not for FIGO IA)	Retrospective cohort study	[77]
	Lymphadenectomy does not decrease the risk of death or disease recurrence compared with no lymphadenectomy	Cochrane systematic review	[78]
	Pelvic lymphadenectomy was associated with increased survival compared with no lymphadenectomy (five-year survival 91.4% vs. 87.3%)	Matched cohort analysis	[79]
	Para-aortic lymphadenectomy was associated with increased survival compared with pelvic lymphadenectomy alone (five-year survival 91.0% vs. 89.8%)	Matched cohort analysis	[79]
	Combined pelvic and paraaortic lymphadenectomy improved survival in intermediate and high-risk EC compared to pelvic lymphadenectomy only (46% decrease in mortality, 13% increase in five-year OS, and 23% increase in five-year DFS)	Meta-analysis	[80,81]
	Removal of >11 pelvic lymph nodes was associated with improved OS and PFS compared with the removal of ≤11 pelvic lymph nodes in grade 3 EC; this association was not observed for grade 1 and 2 EC	Retrospective	[84,85]
	SLNB more accurately detects positive pelvic nodes than systematic lymphadenectomy	Systematic review and meta-analysis	[92]
	SNLB has excellent accuracy in detecting metastases for EC FIGO stage I of all grades and histopathological subtypes (97.2% sensitivity and 99.6% negative predictive value) [99]	Multicenter, prospective cohort study	[97]
	SNLB has excellent accuracy in detecting metastases for high-risk early stage (FIGO I-II) EC (98% sensitivity and 99.5% negative predictive value)	Prospective non-randomized trial	[98]
	In patients with detected positive lymph nodes, performing only SNLB vs. systematic lymphadenectomy did not alter the prognosis	Systematic review and meta-analysis	[92]
**FIGO IIIC**	Complete resection of macroscopic nodal disease improves DFS (37.5 vs. 8.8 months, respectively)	Retrospective	[106]
	Complete resection of macroscopic nodal disease improves survival (five-year disease-specific survival of 63% for completely resected nodal disease versus 43% with macroscopic residual disease)	Retrospective	[108]
	Para-aortic lymphadenectomy in addition to pelvic lymphadenectomy increased PFS and OS (five-year PFS 36% vs. 76%, five-year OS 42% vs. 77%)	Retrospective	[105]
	Systematic pelvic and para-aortic lymphadenectomy did not improve survival vs. SNLB in patients with microscopic nodal metastases (>0.2 mm) only	Retrospective	[110]

**Table 4:** Sites of endometrial cancer recurrences [133].

Type of Recurrence		Rate (%)
**Single-pathway recurrence**		**75.8**
	**Locoregional recurrence**	**15.2**
	Vaginal vault	7.1
	Pelvic	8.1
	**Nodal recurrence**	**13.2**
	Inguinal and intraabdominal	9.1
	Extraabdominal	4.1
	**Distant organ recurrence**	**32.8**
	Single organ	16.2
	Multiple organ	16.6
	**Carcinomatosis recurrence**	**14.6**
**Multiple-pathway recurrence**		**24.2**
**Overall**		**18.2**

**Table 5:** The rate of lymph node metastases according to the molecular profile of EC [157,158,159,160,161].

Molecular Subtype	POLEmut	MMRd/MSI	NSMP	p53abn	Total
Rate of pelvic +/− paraaortic lymph node metastases	0–14%	6–18%	11–19%	18–45%	11–21%
